# Correction: Selective decontamination of the digestive tract in esophagectomy and the incidence of pneumonia and anastomotic leakage: A systematic review and meta-analysis

**DOI:** 10.1371/journal.pone.0338216

**Published:** 2025-12-05

**Authors:** Sander Du Xi Oei, Jasper Gerrit Jan Verbruggen, Sanne Elisabeth Hoeks, Marcus Paulus Buise

The first author’s name is spelled incorrectly. The correct name is: Sander Du Xi Oei. The correct citation is: Oei SDX, Verbruggen JGJ, Hoeks SE, Buise MP (2025) Selective decontamination of the digestive tract in esophagectomy and the incidence of pneumonia and anastomotic leakage: A systematic review and meta-analysis. PLoS One 20(6): e0325241. https://doi.org/10.1371/journal.pone.0325241.
