# Supplementary material for: Selective decontamination of the digestive tract in esophagectomy and the incidence of pneumonia and anastomotic leakage: A systematic review and meta-analysis
Source: PLoS One. 2025 Jun 25;20(6):e0325241. doi: 10.1371/journal.pone.0325241 (PMC12192084; doi:10.1371/journal.pone.0325241)
Supplement: S2 File — (DOCX) [file pone.0325241.s002.docx]

**Selective decontamination of the digestive tract in esophagectomy and the incidence of pneumonia and anastomotic leakage. A systematic review and meta-analysis**

SDX Oei^1¶^, JGJ Verbruggen^1¶^, SE Hoeks^1^, MP Buise^*2^

1. Department of Anesthesiology, Erasmus University Medical Center, Rotterdam, the Netherlands.
2. Department of Anesthesiology, Maastricht University Medical Center, Maastricht, the Netherlands

*** Corresponding author**

**E-mail:** [**marc.buise@mumc.nl**](mailto:marc.buise@mumc.nl) **(MB)**

¶ These authors contributed equally to this work

**Supplementary appendix**

**Supplementary Information 2 (S2):** Search Strategy

A literature search was conducted on 27-02-2022 and on 26-06-2023. Results were combined after removing duplicates.

**Search 27-02-2022:**

| **Database searched** | **Platform** | **Years of coverage** | **Records** | **Records after duplicates removed** |
| --- | --- | --- | --- | --- |
| Embase | Embase.com | 1971 - Present | 27 | 27 |
| Medline ALL | Ovid | 1946 - Present | 17 | 4 |
| Web of Science Core Collection* | Web of Knowledge | 1975 - Present | 17 | 5 |
| Cochrane Central Register of Controlled Trials | Wiley | 1992 - Present | 8 | 1 |
| Additional Search Engines: Google Scholar | | | 139 | 113 |
| **Total** | | | **208** | **150** |

*Science Citation Index Expanded (1975-present) ; Social Sciences Citation Index (1975-present) ; Arts & Humanities Citation Index (1975-present) ; Conference Proceedings Citation Index- Science (1990-present) ; Conference Proceedings Citation Index- Social Science & Humanities (1990-present) ; Emerging Sources Citation Index (2005-present)

**Search 26-06-2023:**

| **Database searched** | **Platform** | **Years of coverage** | **Records** | **Records after duplicates removed** |
| --- | --- | --- | --- | --- |
| Embase | Embase.com | 1971 - Present | 28 | 28 |
| Medline ALL | Ovid | 1946 - Present | 17 | 4 |
| Web of Science Core Collection* | Web of Knowledge | 1975 - Present | 19 | 6 |
| Cochrane Central Register of Controlled Trials | Wiley | 1992 - Present | 10 | 6 |
| Additional Search Engines: Google Scholar | | | 50 | 42 |
| **Total** | | | **124** | **86** |

*Science Citation Index Expanded (1975-present) ; Social Sciences Citation Index (1975-present) ; Arts & Humanities Citation Index (1975-present) ; Conference Proceedings Citation Index- Science (1990-present) ; Conference Proceedings Citation Index- Social Science & Humanities (1990-present) ; Emerging Sources Citation Index (2005-present)

**Search 27-08-2024:**

| **Database searched** | **Platform** | **Years of coverage** | **Records** | **Records after duplicates removed** |
| --- | --- | --- | --- | --- |
| Embase | Embase.com | 1971 - Present | 29 | 29 |
| Medline ALL | Ovid | 1946 - Present | 18 | 4 |
| Web of Science Core Collection* | Web of Knowledge | 1975 - Present | 19 | 6 |
| Cochrane Central Register of Controlled Trials | Wiley | 1992 - Present | 11 | 5 |
| Additional Search Engines: Google Scholar | | | 50 | 44 |
| **Total** | | | **127** | **88** |

*Science Citation Index Expanded (1975-present) ; Social Sciences Citation Index (1975-present) ; Arts & Humanities Citation Index (1975-present) ; Conference Proceedings Citation Index- Science (1990-present) ; Conference Proceedings Citation Index- Social Science & Humanities (1990-present) ; Emerging Sources Citation Index (2005-present)

**Embase.com**

('esophagus surgery'/exp OR ('esophagus'/exp AND ('surgery'/de OR 'surgery':lnk)) OR (esophagoplast* OR oesophagoplast* OR ((esophag* OR oesophag*) NEAR/3 (anastomos* OR resect* OR surg*)) OR oesophagectom* OR esophagectom* OR ivor-lewis* OR mckeown* OR transhiatal*):ab,ti,kw) **AND** ('selective digestive decontamination'/de OR (((selective*) AND (decontamination*) AND (digestive* OR oropharyng*)) OR SDD OR SOD):ab,ti,kw)

**Medline (Ovid)**

(Esophagectomy/ OR Esophagoplasty/ OR (Esophagus/ AND (surgery.fx. OR Surgical Procedures, Operative/)) OR (esophagoplast* OR oesophagoplast* OR ((esophag* OR oesophag*) ADJ3 (anastomos* OR resect* OR surg*)) OR oesophagectom* OR esophagectom* OR ivor-lewis* OR mckeown* OR transhiatal*).ab,ti,kf.) **AND** ((((selective*) AND (decontamination*) AND (digestive* OR oropharyng*)) OR SDD OR SOD).ab,ti,kf.)

**Web of Science**

TS=(((esophagoplast* OR oesophagoplast* OR ((esophag* OR oesophag*) NEAR/2 (anastomos* OR resect* OR surg*)) OR oesophagectom* OR esophagectom* OR ivor-lewis* OR mckeown* OR transhiatal*)) **AND** ((((selective*) AND (decontamination*) AND (digestive* OR oropharyng*)) OR SDD OR SOD)))

**Cochrane Central**

((esophagoplast* OR oesophagoplast* OR ((esophag* OR oesophag*) NEAR/3 (anastomos* OR resect* OR surg*)) OR oesophagectom* OR esophagectom* OR ivor NEXT lewis* OR mckeown* OR transhiatal*):ab,ti,kw) **AND** ((((selective*) AND (decontamination*) AND (digestive* OR oropharyng*)) OR SDD OR SOD):ab,ti,kw)

**Google Scholar**

esophagoplasty|oesophagoplasty|"esophagus|oesophagus|esophageal|oesophageal anastomosis|resection|resected|surgery|surgical|surgeries"|oesophagectomy|esophagectomy|ivor-lewis|mckeown|transhiatal "selective decontamination digestive"|SDD
