# Supplementary material for: Selective decontamination of the digestive tract in esophagectomy and the incidence of pneumonia and anastomotic leakage: A systematic review and meta-analysis
Source: PLoS One. 2025 Jun 25;20(6):e0325241. doi: 10.1371/journal.pone.0325241 (PMC12192084; doi:10.1371/journal.pone.0325241)
Supplement: S3 File — (DOCX) [file pone.0325241.s003.docx]

**Supplementary Information 3. Funnel plots.**


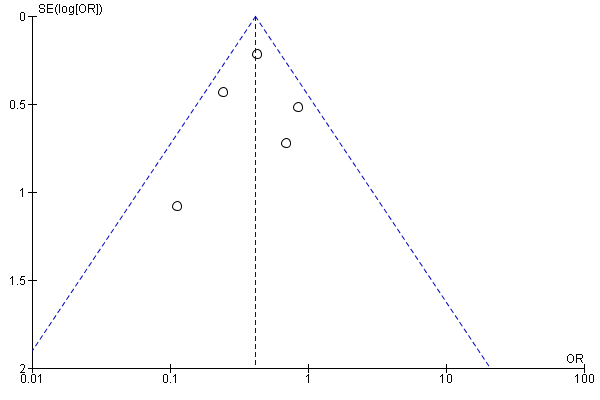


Funnel plot of comparison: 1 Meta analysis, outcome: 1.1 Postoperative pulmonary complications.


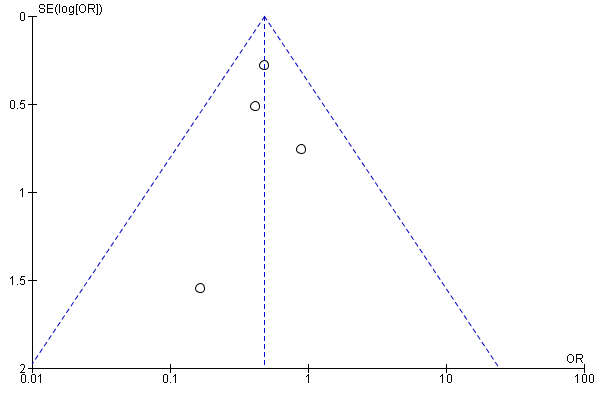


Funnel plot of comparison: 1 Meta analysis, outcome: 1.2 Anastomotic leakage.
