# Supplementary material for: Selective decontamination of the digestive tract in esophagectomy and the incidence of pneumonia and anastomotic leakage: A systematic review and meta-analysis
Source: PLoS One. 2025 Jun 25;20(6):e0325241. doi: 10.1371/journal.pone.0325241 (PMC12192084; doi:10.1371/journal.pone.0325241)
Supplement: Attachment — Submitted filename: Rebuttal letter PLOS ONE.docx [file pone.0325241.s005.docx]

**Rebuttal letter PLOS ONE**

Sander Oei
Erasmus Medical Center
Dr. Molewaterplein 40, 3015 GD

Rotterdam, The Netherlands

05-09-2024

Dear Editors,

We thank the reviewers for their comment on the manuscript (PONE-D-24-34131) and have edited the manuscript to address their concerns.

We understand that PLOS ONE wishes to provide up-to-date overviews and have renewed our literature search to meet this requirement.

We believe that the manuscript is now suitable for publication in PLOS ONE.

Sincerely,

Sander Oei

On behalf of all authors.
