# Supplementary material for: Selective decontamination of the digestive tract in esophagectomy and the incidence of pneumonia and anastomotic leakage: A systematic review and meta-analysis
Source: PLoS One. 2025 Jun 25;20(6):e0325241. doi: 10.1371/journal.pone.0325241 (PMC12192084; doi:10.1371/journal.pone.0325241)
Supplement: Attachment — Submitted filename: Response to Reviewers.docx [file pone.0325241.s006.docx]

**Response to Reviewers**

Sander Oei
Erasmus Medical Center
Dr. Molewaterplein 40, 3015 GD

Rotterdam, The Netherlands

13-02-2025

Dear Editorial Board,

**Subject: Rebuttal of Manuscript Selective decontamination of the digestive tract in esophagectomy and the incidence of pneumonia and anastomotic leakage. A systematic review and meta-analysis**

We are writing in response to the comments and suggestions provided by the reviewers regarding our manuscript titled “Selective decontamination of the digestive tract in esophagectomy and the incidence of pneumonia and anastomotic leakage. A systematic review and meta-analysis”.

We sincerely appreciate the time and effort that the reviewers have dedicated to evaluating our work. We have carefully considered their comments and have made several revisions to the manuscript to address their concerns. Below, we provide a detailed point-by-point response to each of the reviewers’ comments. We hope that our adjustments can resolve any ambiguities and alleviate concerns regarding our research.

**Review Comments to the Author** 

Reviewer #1: Oei and colleagues from the Netherlands present a systematic review regarding the use of selective gut decontamination for reducing pneumonia and leak after oeospahgectomy. Over the years it has been challenging to gain traction in these areas with non-operative methods, mainly because of the surgical technique has been evolving, and the highly multifactorial nature of post-oesophagectomy pneumonia / acute lung injury and leaks. Overall it seems a well-executed review and thoughtfully written. The quality of the input studies are not great but I agree some insight comes from them. I couldn’t find much typographically error. It seems the goal is to develop a randomised study targeting the microbiome to reduce risk, which I would probably support given where we are now surgically (and collaboratively) 
 
I’ve got a few comments for the authors that when addressed would strengthen the paper; 
**1. The key challenge you have in justifying an RCT is that reducing these risks seems to have no bearing at all on ITU stay or LOS. In some studies both were slightly longer in SDD.**

Response:

Thank you for your thoughtful response. We believe that an important point is raised here, highlighting the need to consider patient care as a comprehensive continuum. The studies we reviewed do not show a clear benefit in terms of the length of intensive care unit stay or total hospital stay with the use of selective decontamination of the digestive tract (SDD) following esophagectomy. However, there is an observable effect on pneumonia and anastomotic leak, which were our primary outcomes. While the reduction in these outcomes may not have an immediately visible effect on ICU stay duration, it could still be beneficial for patients in the long term by reducing complications, for example fewer additional surgeries due to anastomotic leaks. If shortening patient hospital stays is a priority, it could be valuable to conduct a study specifically focused on identifying the key components contributing to length of stay. We have addressed this in the sixth paragraph of our discussion on page 14 of the manuscript. We hope this sufficiently addresses your comment.

**2. In the introduction please provide some description regarding SDD. Is it oral or IV? Is it one dose or a week? How does it differ to the standard ‘on-induction’ IV doses. Understood there is a partial description in table 1**

Response:

Thank you for your comment. This could indeed have been described more clearly in the article. We have added an additional paragraph in the introduction describing the route of administration of SDD. We hope that this, in combination with Table 1, provides sufficient clarification.

Introduction, page 4:

“These antibiotics are most commonly administered both orally and via a gastric tube, if present. The non-absorbable antibiotics—polymyxin E and tobramycin target a broad spectrum of potentially harmful aerobic Gram-negative rods and S. aureus. Amphotericin B is often added to prevent the overgrowth of molds and yeasts [20].”

**3. Every centre I have worked in provides 24-48h of antibiotic coverage for the operation and immediate post-op. How does the proposed strategies differ, and how has the IV prescription been considered in the presented studies (your description of the controls could be improved)**

Response:

Thank you for your comment. The description of the difference between standard perioperative antibiotic use and SDD was indeed not sufficiently clear. We have clarified how the control group protocols differ from those in the SDD group in our manuscript.

Introduction, page 4:

“SDD differs from standard perioperative antibiotic prophylaxis, which is typically administered intravenously at the time of induction and generally lasts for a shorter duration (e.g., a single-dose administration during surgery or 24-48 hours of postoperative coverage).”

**4. Because the antibiotics regimes are not well described, it is difficult to judge the risk of adverse events like diarrhoea, c. diff, carbapenamase producing infections etc. These aren’t reported. I disagree with the line that there is very little ill-effect from SDD use.**

Response:

Thank you for your comment. We have reviewed how we phrased the statement that SDD use carries minimal risks. While SDD is more commonly used in the Netherlands, the assertion that it carries very few risks may have been overly assertive. We have added references to studies that support our statement, but we have also revised the text to better nuance our statement.

Introduction, page 4:

“Selective decontamination of the digestive tract (SDD) has been associated with improved patient outcome in critically ill patients [20]. A systematic review of perioperative SDD in elective gastrointestinal surgery suggested that a combination of perioperative SDD and perioperative intravenous antibiotics reduces the rate of postoperative infection and anastomotic leakage compared with systemic antibiotic prophylaxis alone [21].

Because micro-aspirations may often occur after esophagectomy, treatment with SDD in this patient group is of particular interest [22, 23].”

**5. The discussion could be improved by checking the oesophageal microbiome literature for how these decontamination strategies actually change diversity**

Response:

Thank you for your comment. In the second-to-last paragraph of our discussion, we have included a summary of the literature we found regarding the microbiome of the oesophagus and SDD use. Although it is not clear how the esophageal microbiome is altered by SDD, it appears to be important, as demonstrated by studies conducted in colon and pancreatic surgery.

Discussion, page 14-15:

*“*The composition of different antibiotics used as components of SDD was not uniform in our included studies. We are therefore unable to make a statement about the ideal composition of SDD. The esophageal microbiome, which is believed to play a significant role in the development of postoperative complications, is influenced by the oral flora and has associations with various diseases, including esophageal cancer [51, 52]. While previous studies have highlighted the important role of the microbiome in colon surgery, and research in pancreatic surgery has shown that the microbiome is altered by SDD, the effect of SDD on the diversity of the esophageal microbiome remains unclear [53, 54]. Further investigation into how SDD impacts microbial diversity in the esophagus is warranted.*”*

**6. It would be worth pointing out that with advancing surgical technique (minimally invasive surgery, sparing of the pulmonary vagal branches, two lung ventilation, ERAS, prehabilitation) many of the precipitants of PPC have been considerably decreased, so potentially aspiration may have a proportionally bigger effect on acute lung injuries than when the first of the included studies were performed.**

We believe this is a very important comment and a valuable addition to our discussion. Thank you for this remark. In the third and fourth paragraphs of our discussion, we have provided a clearer explanation of recent developments and how we believe these should be considered when interpreting our outcomes. A potentially greater role for aspiration seems plausible.

Discussion, page 12:

“Although patient care has changed over the years, including the introduction of new minimally invasive surgical techniques, sparing of the pulmonary vagal branches, Enhanced Recovery After Surgery (ERAS) and prehabilitation the mechanisms of postoperative pneumonia and AL (micro-aspirations and tissue perfusion) remain unchanged [10, 34-39].

Among the strategies mentioned, minimally invasive surgery, ERAS, and prehabilitation are well-established in routine clinical practice. The anticipated beneficial effects of vagal-sparing surgical techniques are attributed to the vagus nerve's role in modulating anti-inflammatory responses [40, 41]. However, this technique remains contentious, and its application is currently limited to a small cohort of patients with early-stage cancers or severe epithelial dysplasia.”

**7. Following on from the previous point, at our centre we still preformed contrast studies to check for aspiration during swallowing, and delayed gastric conduit emptying. Our pneumonia rate is low. Thus if aspiration is considered to be the pathological mechanism, it would be good to understand why it is happening and treat the cause, rather than just treat the effects. This would need to be accounted for in a subsequent randomised studies** 

Response:

Thank you for this addition. In our hospital, contrast studies are not routinely performed, and routine upper gastrointestinal contrast studies are not currently recommended in the ERAS guidelines, however, we do observe in the literature that it may be a promising approach to investigate this. We have included this as an addition in our discussion.

Discussion, page 13:

“Swallowing dysfunction and tracheobronchial aspiration occur in a significant number of patients after esophagectomy. The mechanism is thought to be related to swallowing dysfunctions, possibly exacerbated by recurrent laryngeal nerve palsy [23, 42]. Silent aspiration occurs in 44.7% of patients who had been confirmed as having aspiration with videofluoroscopic swallowing studies [43]. Furthermore, the anatomic location of the gastric conduit may play an etiological role in aspiration. Bulging of the gastric conduit into the right chest likely delays gastric emptying leading to a higher rate of lesions on computed tomography [44]. The management of delayed gastric emptying remains a topic of ongoing debate [45]. Routine upper gastrointestinal contrast studies are not currently recommended in the ERAS guidelines, although their use in identifying patients with delayed gastric conduit emptying shows promise [37, 46].”

**Minor:** 
**1. Page 3 last four lines “Bacteria may interfere…” these lines are not well balanced. Undoubtedly there will be helpful bacteria also**

Response:

We agree that we had provided a somewhat vague description of bacteria in general, as not all bacteria cause issues. We have revised the text to provide a more specific and accurate description

Introduction, page 3:

“An additional factor that possibly contributes to anastomotic leakage is the presence of bacteria that produce endo- or exotoxins. Certain bacteria, such as P. aeruginosa, may interfere with the healing of the anastomosis and cause anastomotic leakage through downregulation of fibroblast growth, production of toxins, and infection, which lead to necrosis (also promoted via microcirculatory disturbances) [17].”

**2. Page 12 line 14 -16 non-significant lower risk please revise this to “the risk of anastomatic leakage in randomised trials was not significantly different”.**

Response:

Thank you for the improvement. This is a more accurate way to describe the statistics, and we have made the necessary adjustment.

**Reviewer #2:** It is currently unknown whether selective decontamination of the digestive tract (SDD) affects incidence of postoperative pneumonia and anastomotic leakage in patients undergoing esophagectomy. The authors summarized current evidence regarding SDD in patients undergoing esophagectomy. Through searching various databases, they found that although the data indicates that using SDD in patients undergoing an esophagectomy was associated with a lower incidence of postoperative pneumonia and anastomotic leakage, the available studies were not of sufficient quality to make a recommendation, given their age and risk of bias. A high-quality randomized controlled trial using standardized outcome definitions is needed to substantiate claims about SDD use in esophagectomy. 
 
Overall, the manuscript is well-written and easy follow. A few concerns need to be addressed before publishing. 

**1. Did the authors include Scopus database?**

Response:

Thank you for your question. This is an important point regarding the completeness of our review. The search was developed by experienced medical librarians. Although we did not use Scopus in this systematic review, we believe that we have comprehensively covered the relevant literature on the topic. We consulted other extensive and reputable databases, such as Web of Science, Medline, Cochrane, Embase, and Google Scholar which provide broad coverage of the medical and scientific literature. These databases are highly regarded in our field and have significant overlap with Scopus. Given their comprehensive nature and the inclusion of high-quality, peer-reviewed articles, we are confident that our literature search was exhaustive and that the important studies were identified.

**2. The selection criteria should be presented in details.**

Thank you for your comment regarding the description of our selection criteria, which we may not have clearly articulated earlier in our manuscript. We aimed to use a broad search strategy to ensure that no studies were missed. Therefore, we included every study involving esophagectomy in combination with SDD, excluding only case series and case reports. No further restrictions were applied, and we manually reviewed all studies for their relevance to our review. We hope this process is now clearly described in our manuscript under the 'Selection Criteria' section.

Methods, page 4, 5:

“Databases were searched for articles on esophagectomy, regardless of the surgical technique used or the year of publication. To obtain a comprehensive view of the literature that could be effectively combined in a meta-analysis, we included both observational and interventional studies in the search, while excluding case series and case reports. The studies had to report on postoperative pulmonary complications or anastomotic leaks to be included, which were the primary outcomes of interest for our review

Databases were searched for articles on esophagectomy, regardless of surgical technique, that examined the use of SDD and compared occurrence of pneumonia or anastomotic leakage. Both observational and interventional studies were included in the search, case series and case studies were excluded.”

**3. Strength and limitations should be included in the Discussion section.**

**Response:**

Thank you for pointing out that our strengths and limitations were not clearly highlighted in our discussion. While we are proud of our comprehensive search strategy, which provides a good overview of the literature, we have now more clearly articulated the gaps that limit our study. These include the fact that some articles are outdated, all studies may be subject to some form of bias, and that outcome assessments are not consistently defined. We hope that these points are now more clearly outlined throughout the discussion.

We think that the revisions we made in response to the reviewers’ comments have improved the manuscript. We would like to express our sincere gratitude for the valuable feedback, and we hope that the revised manuscript will now meet the standards of PLOS ONE.

We are looking forward to your consideration of our revised submission and are happy to provide any further clarifications if needed.

Sincerely,

Sander Oei

On behalf of all authors.
