# Supplementary material for: Selective decontamination of the digestive tract in esophagectomy and the incidence of pneumonia and anastomotic leakage: A systematic review and meta-analysis
Source: PLoS One. 2025 Jun 25;20(6):e0325241. doi: 10.1371/journal.pone.0325241 (PMC12192084; doi:10.1371/journal.pone.0325241)
Supplement: Attachment — Submitted filename: Response_to_Reviewers_auresp_2.docx [file pone.0325241.s007.docx]

**Response to Reviewers**

Sander Oei
Erasmus Medical Center
Dr. Molewaterplein 40, 3015 GD

Rotterdam, The Netherlands

20-04-2025

Dear Editorial Board,

**Subject: Rebuttal of Manuscript Selective decontamination of the digestive tract in esophagectomy and the incidence of pneumonia and anastomotic leakage. A systematic review and meta-analysis**

We are writing in response to the comments and suggestions provided by the reviewers regarding our manuscript titled “Selective decontamination of the digestive tract in esophagectomy and the incidence of pneumonia and anastomotic leakage. A systematic review and meta-analysis”.

We sincerely appreciate the time and effort that the reviewers have dedicated to evaluating our work. We have carefully considered their comments and have made several revisions to the manuscript to address their concerns. Below, we provide a detailed point-by-point response to each of the reviewers’ comments. We hope that our adjustments can resolve any ambiguities and alleviate concerns regarding our research.

Journal Requirements:
Please review your reference list to ensure that it is complete and correct. If you have cited papers that have been retracted, please include the rationale for doing so in the manuscript text, or remove these references and replace them with relevant current references. Any changes to the reference list should be mentioned in the rebuttal letter that accompanies your revised manuscript. If you need to cite a retracted article, indicate the article’s retracted status in the References list and also include a citation and full reference for the retraction notice.

**Response:**

We have reviewed and updated our reference list accordingly. Two studies were inadvertently listed twice:

1. Riedl, S., et al. Microbiological and clinical effects of selective bowel decontamination in transthoracic resection of carcinoma of the esophagus and cardia. Chirurg, 2001, 72(10): 1160–1170.
2. Myburgh, J.A., et al. Effect of Selective Decontamination of the Digestive Tract on Hospital Mortality in Critically Ill Patients Receiving Mechanical Ventilation: A Randomized Clinical Trial. JAMA, 2022, 328(19): 1911–1921.

We have corrected this duplication and ensured that the reference list is complete and up to date. No retracted articles are cited in the current version.

**PACE:**

While revising your submission, please upload your figure files to the Preflight Analysis and Conversion Engine (PACE) digital diagnostic tool, <https://pacev2.apexcovantage.com/>. PACE helps ensure that figures meet PLOS requirements. To use PACE, you must first register as a user. Registration is free. Then, login and navigate to the UPLOAD tab, where you will find detailed instructions on how to use the tool. If you encounter any issues or have any questions when using PACE, please email PLOS at [figures@plos.org](mailto:figures@plos.org). Please note that Supporting Information files do not need this step.

**Response:**

All figures that were submitted last time were uploaded through PACE.

6**. Review Comments to the Author**

Reviewer #1: Thanks for your work

Please double-check the figure / table legends before finalising

Also please delete this sentence from the discussion which i think is non-contributory: "Among the strategies mentioned, minimally invasive surgery, ERAS, and prehabilitation are wellestablished in routine clinical practice. The anticipated beneficial effects of vagal-sparing surgical techniques are attributed to the vagus nerve's role in modulating anti-inflammatory responses [40, 41]. Formatted: Heading 1, Line spacing: single 14
However, this technique remains contentious, and its application is currently limited to a small cohort of patients with early-stage cancers or severe epithelial dysplasia."

**Response**

We have carefully reviewed all figure and table legends and revised them to better align with the PLOS ONE submission guidelines. If there are any specific elements that still require attention, we would be grateful for further guidance.

As suggested, we have removed the sentence from the Discussion section along with the two associated references.

Lastly:

We removed lines 106–109, as they were redundant and repeated information already provided in the preceding sentences.

We think that the revisions we made in response to the reviewers’ comments have improved the manuscript. We would like to express our sincere gratitude for the valuable feedback, and we hope that the revised manuscript will now meet the standards of PLOS ONE.

We are looking forward to your consideration of our revised submission and are happy to provide any further clarifications if needed.

Sincerely,
Sander Oei
On behalf of all authors.
